# Space Subdivision in Indoor Mobile Laser Scanning Point Clouds Based on Scanline Analysis

**DOI:** 10.3390/s18061838

**Published:** 2018-06-05

**Authors:** Yi Zheng, Michael Peter, Ruofei Zhong, Sander Oude Elberink, Quan Zhou

**Affiliations:** 1 Beijing Advanced Innovation Center for Imaging Technology, College of Resource Environment and Tourism, Capital Normal University, Beijing 100048, China; 2150902027@cnu.edu.cn (Y.Z.); 2150902043@cnu.edu.cn (Q.Z.); 2 Faculty of Geo-Information Science and Earth Observation, University of Twente, P.O. Box 217, 7514 AE Enschede, The Netherlands; m.s.peter@utwente.nl (M.P.); s.j.oudeelberink@utwente.nl (S.O.E.)

**Keywords:** opening detection, space subdivision, trajectory, indoor point clouds

## Abstract

Indoor space subdivision is an important aspect of scene analysis that provides essential information for many applications, such as indoor navigation and evacuation route planning. Until now, most proposed scene understanding algorithms have been based on whole point clouds, which has led to complicated operations, high computational loads and low processing speed. This paper presents novel methods to efficiently extract the location of openings (e.g., doors and windows) and to subdivide space by analyzing scanlines. An opening detection method is demonstrated that analyses the local geometric regularity in scanlines to refine the extracted opening. Moreover, a space subdivision method based on the extracted openings and the scanning system trajectory is described. Finally, the opening detection and space subdivision results are saved as point cloud labels which will be used for further investigations. The method has been tested on a real dataset collected by ZEB-REVO. The experimental results validate the completeness and correctness of the proposed method for different indoor environment and scanning paths.

## 1. Introduction

Humans currently perform most of their activities in indoor spaces, such as work spaces and sports facilities, which suggests the great potential of indoor scenes for broad applications, such as indoor modelling and navigation [1,2]. To meet the demands of these applications, many scanners, such as terrestrial laser scanners and RGB-D sensors, have been widely used to acquire 3D point clouds in indoor environments. In recent years, indoor mobile laser scanners (IMLS) have emerged as the most versatile technology for indoor mapping because of their relatively high accuracy and resolution, portability, easy access and high acquisition speed. In addition to providing point clouds, such systems also provide a continuous trajectory of the device’s location.

Man-made indoor spaces are subdivided into several smaller spaces by walls and doors, and indoor space subdivision is performed to make smaller datasets to improve processing efficiency and acquire specific semantic information essential for modeling [3,4] and indoor navigation [2]. However, there is no information subdividing the different parts of the indoor space since the scanning devices cannot directly distinguish the points that belong to different spaces [3,5]. Several methods have been proposed to subdivide indoor space by analyzing vertical planar patches [6], trajectories [7], 2.5D models [8], etc. However, these methods are based on entire dataset, which leads to complicated operations, high computational loads and low processing speeds.

**Figure 1:**
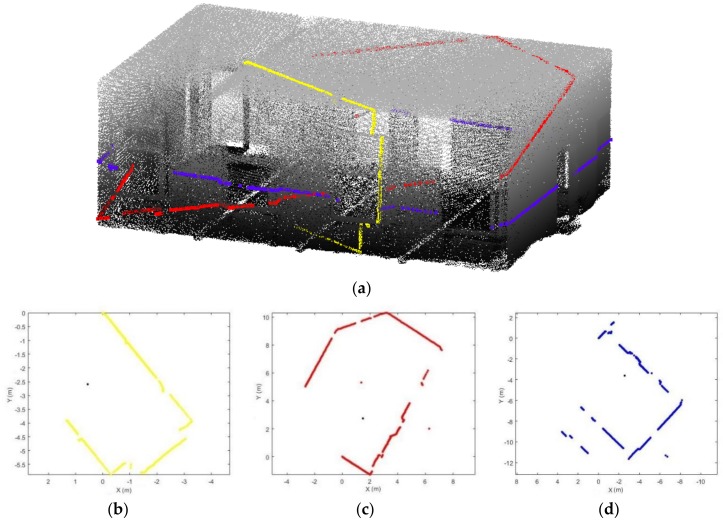
Example scan pattern of three scanlines in a room: (**a**) 3D view of scanlines; (**b**–**d**) corresponding 2D scanlines (different colour represents different scanlines, black point: scanner position).

In mobile laser scanning system, scanlines are generated in one scan direction by a rotating mirror which is used for laser beam deflection (as shown in Figure 1b–d). Complete 3D point clouds can be generated by the scanlines and it corresponding scanner attitude [9]. Processing based on a scanline showcase can be executed comparatively fast and uncomplicatedly compared with the analysis of an entire dataset [10,11]. During scanning, the scanner captures each scanline, which could allow direct processing, thus these methods are applicable for online data preparation for subsequent analysis. One problem associated with using single scanlines is that limited information is provided for the recognition of indoor scenes because in certain orientations, a scene is represented by an internal contour, which is composed of a set of points. To solve this problem, we try to join the analysis of the scanner trajectory and the other scanlines to obtain fine details of indoor objects. To date, several algorithms have been proposed for the detection of indoor objects based on trajectories [4,12,13]. However, these methods identify the trajectory shape, which has limited capacity to discriminate objects that do not interact with the trajectory, such as doors passed through by the operator. The goal of this research is to detect openings based on a scanline analysis and subdivide space by a joint analysis of trajectories and point clouds. Our method uses a set of rules to extract pairs of points as opening candidates and performs buffer operations to extract optimal openings. Then, a two-step space subdivision method based on the extracted openings is described. First, the trajectory points are subdivided into different spaces based on doors extracted from the intersection between the trajectory and detected openings. Second, the corresponding point clouds related to the trajectory points within the same space are subdivided based on the extracted doors. This approach can be applied to real-time processing, simultaneous localization and mapping (SLAM), mobile robots, etc. The remainder of this article is divided as follows: Section 2 of this paper introduces related works. Section 3 describes the instruments and datasets used. Section 4 reports each step of the methodology in detail. Section 5 presents the results and discussion. Section 6 concludes the paper and provides an outlook on future work.

## 2. Related Work

As mentioned above, the proposed methodology starts with a scanline analysis. Hebel and Stilla [11] used a real-time capable filter operation that based on random sample consensus to distinguish clutter and men-made objects. In another study, Hu and Ye [10] proposed a Douglas-Peucker algorithm for segmenting the scanline into segment objects based on height variation and set a simple rule-based classification to distinguish building and non-building in outdoor environment. The authors claim the method is still needed 2D or 3D neighbourhood to meet higher detection quality. However, these proposed methods are too simple to be appropriate for the analysis of indoor scenes which are often complex and cluttered environments. In indoor space, the authors in [7,13] proposed a supervised learning algorithm to detect humans or classify different indoor spaces. Borrmann [14] calls the virtual edges between disconnected parts in scanline “jump edges” and presents a method that uses the jump edges to separate explored and unexplored regions of the environment. Nevertheless, the scanlines that are analysed by most of the current proposals are acquired by the scanner which are fixedly mounted on the scanning system. In this work, we address the challenge of analysis the scanlines that captured by the moving laser scanner head since the scanlines acquired in different scanner attitude presents different point pattern and information of indoor space (as shown in Figure 1b–d).

Regarding the detection of openings, A pipeline of techniques used to extract closed doors based on orthoimages was proposed by Díaz-Vilariño et al. [15]. Another door detection method that uses both geometric and colour information to detect open doors and closed doors was proposed by Quintana et al. [16] These methods added colour information on consideration. Nikoohemat et al. presented methods that use the combinations of voxels and trajectories to detect open and closed doors [5]. Some other authors have worked on window detection. For example, Tuttas and Stilla [17] used a Fourier Transform to detect windows based on points lying behind the detected indoor facade planes. Their approaches should use all the dataset for processing which have weakness like high computational loads. In addition, a Markov random field (MRF) framework to automatically identify windows was proposed in [18]. To acquire both windows and doors in indoor environments, an opening extraction method using graph-cuts to process point clouds under cluttered and occluded environments was demonstrated in [19]. Adan and Huber [20] used a support vector machine classifier to detect openings by learning a model with the size, shape, and location of openings. However, these methods need training the dataset for acquire important parameters in methodology.

A body of research has also focused on space subdivision. Nikoohemat et al. [5] used the concept of volumetric empty space by applying voxel space to partition empty spaces based on opening and wall detection results. Mura et al. [6,21] applied a diffusion process to space partitioning induced by the candidate walls to extract individual rooms. Armeni et al. [22] used a detection-based semantic parsing method to parse point clouds into their constituents. Turner et al. [8,23] used a graph-cut approach to partition space into separate rooms based on the volumetric partitioning of interior space which is acquired by a Delaunay Triangulation on the plane. Xu et al. [24] used a simplification of 2D floor plan to subdivide the free space inside buildings by applied Delaunay triangulation. However, this method just applied to subdivide space into navigable and non-navigable spaces which aims at being used in path-finding applications. Space subdivision base on Delaunay triangulation are analysing the connection relationships among the nodes in the derived network. The configuration and the size of the indoor spaces will affect the indoor network. Hence, these approaches may entirely ignore some small spaces, like doorways, the shapes of rooms, etc.

## 3. Instruments and Data

Two datasets were used in this research which were captured on different floors of one of the buildings of the Technical University of Braunschweig (Germany). They are part of the datasets of the ISPRS benchmark on indoor modelling [25] which provides more information about the data. The statistics are given in Table 1.

Our input datasets contain the point clouds and the trajectory acquired using ZEB-REVO, a hand-held laser scanner [26]. Trajectory is a set of points that records the movement of scanner system. The trajectory is related to the point cloud by time attribute. The scanner system consists of a 2D laser range scanner, an IMU and a motor drive. The characteristics of ZEB-REVO laser device are shown in Table 2. The laser in this scanning system is a 2D time-of-flight laser with 270 degrees of view, 905 nm laser wavelength, up to a 30 m scanning range and ±30 mm range noise. During the data acquisition process, the 2D scanner head is rotated around the roll axis of the system and scans the indoor environment with 100 scanlines per second. This process leads to a distinct pattern of scanlines as shown in Figure 1.

## 4. Methods

**Figure 2:**
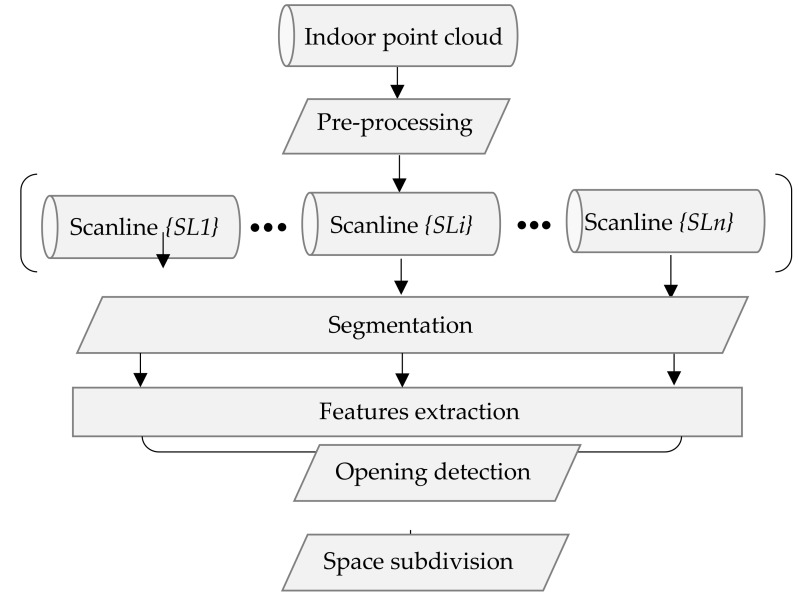
Workflow of proposed methodology.

This section describes the proposed methodology. Section 4.1 introduces the process of data pre-processing, Section 4.2 presents the method of segmentation, and Section 4.3 is devoted to illustrating the detail of extracted features. Then, Section 4.4 describes the space subdivision method. The space subdivision process is illustrated in Section 4.5, and the processes of extracting doors and performing the join analysis of trajectories are presented in detail. The general framework of this research is shown in Figure 2. Pre-processing is a necessary step to restore data to the realistic state observed during data acquisition. Then, segmentation and feature extraction are separately processed on each scanline. Certain points are differentiated, such as the points in an opening area, and then extracted by analysing the features of the segments. To acquire reliable opening detection results, we analyse multiple opening candidates after projecting to the local coordinate system. Then, we use a method that combines the trajectory and extract openings to subdivide the indoor space. Ultimately, the extracted information is saved as point labels that will be used for further investigations.

### 4.1. Pre-Processing

**Figure 3:**
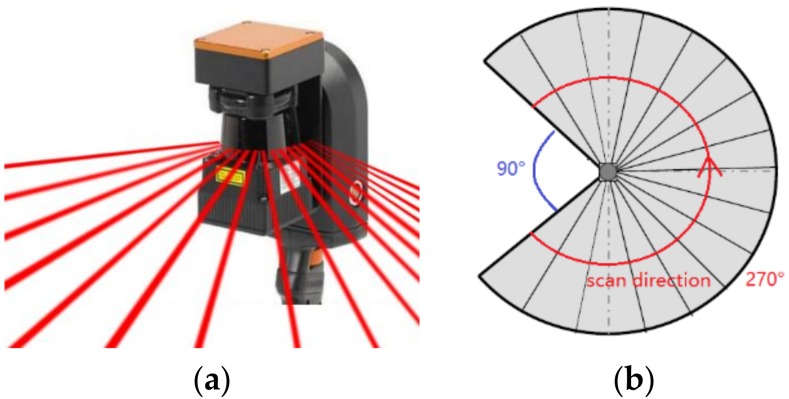
Example of scanning angle in 3D (**a**) and 2D (**b**).

To restore the data to the original state observed during data acquisition, we need to extract scanlines from the entire point cloud by assessing time differences between neighbouring points. The constant rotating scanner will send out a laser beam each time it rotates 0.625° in the same scanline, and it will cease to send out the laser beam within 270° field of view (as shown in Figure 3). Consequently, the time difference value between points within the same scanline is smaller than the time difference between neighbouring scanlines.

However, not all scanlines have exactly 432 points in practice since surfaces that are more than 30 m away from the scanner will not result in a valid signal. This situation may lead to a larger time difference within the same scanline. Accordingly, a threshold $th_{time}$ can be used to determine whether the time difference between two neighbouring points is large enough to separate the point cloud into two scanlines. Once the time difference is higher than the threshold, we conclude that the time stamp is between the time of these two points. At the end of this step, entire point clouds will have been split into several 3D scanlines. The details of threshold $th_{time}$ selection will be described in Section 5.1.

During data acquisition, the scanner first acquires 2D scanlines in the scanner coordinate system and then transfers the coordinates into the local coordinate system using the SLAM algorithm [27]. To acquire the point cloud in the scanner coordinate system, we use a quaternion that represents the scanning orientation to restore the scanline from the local coordinate system into the scanner coordinate system. Suppose that the first point of a scanline in the local coordinate system is *P*_0_, the scanline points in local coordinates is *Pw* [*Xw*, *Yw*, *Zw*], and the corresponding position in the scanner coordinate system is *Psl* [*Xsl*, *Ysl*, *Zsl*]. The rotation matrix *R* is then calculated from the quaternion. The coordinate value of *Psl* can be calculated by Equation (1):

(1) $$\left[\begin{array}{c} Xsl\\ \begin{array}{c} Ysl\\ Zsl \end{array}\\ 1 \end{array}\right]=\left[\begin{array}{cc} R & -RP_{0}\\ 0 & 1 \end{array}\right]\left[\begin{array}{c} Xw\\ Yw\\ \begin{array}{c} Zw\\ 1 \end{array} \end{array}\right],$$

**Figure 4:**
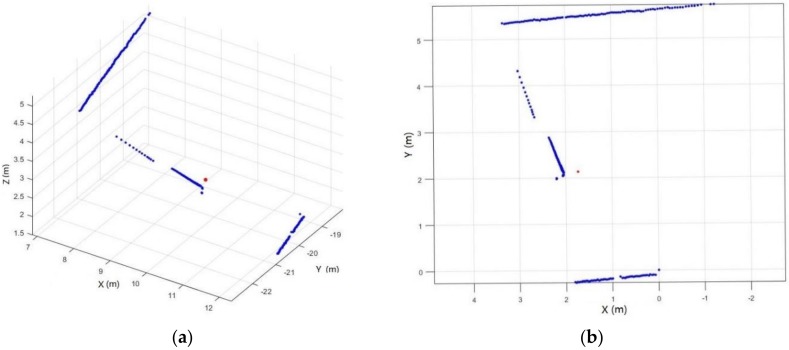
Example scanline before (**a**) and after (**b**) projection (red point: scanner position; blue point: scanline point).

Exemplary results for certain scanlines are shown in Figure 4b.

### 4.2. Segmentation

**Figure 5:**
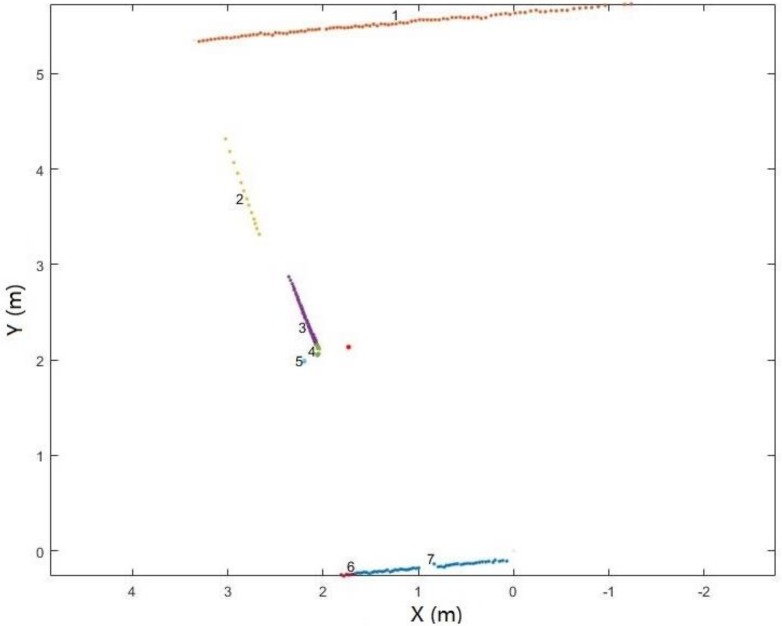
Example of scanline segmentation result (red point: scanner position).

In indoor environments, typical objects, such as ceilings, floors and walls, will be recorded as straight-line segments within the scanline. Compared to the points, segments will carry more stable information relative to analysis of the point distribution in a local neighborhood. Meanwhile, some segment features, such as the line vector, are stable and useful for classification [28]. In this step, the line segmentation method was used to split the 2D single scanline into linear segments. Each single scanline is used as input. This segmentation approach first uses several points to fit a line and accepts it as a candidate if the mean values of a range of residuals is low enough. The following step combines the results of forward and backward processing to produce accurate linear segments and prevent tilted segments [29]. The points will be labelled as belonging to different segments after the segmentation process. Figure 5 demonstrates an example of segmentation result.

### 4.3. Feature Generation

Generating features is an essential task because it can help us gain knowledge about the local environment around a segment. In this section, the features are achieved by analysing the geometric features and local contextual information, which are requested as input sources to build the classifier described in Section 4.4. Suppose that we are given a number of segments 1, …, *i* that are segmented from one scanline. The proposed segment features are separated into two types: segment features and segment pair features. Segment features are derived from an analysis of the point distribution in the single segment, whereas segment pair features describe the relationship between a pair of segments. For each segment *i*, the segment features used here are summarized as follows:

(1) Segment length *Li*: The Euclidean distance between first point and last point of segment *i;*
(2) Segment size *Si*: The number of points in the segment *i;*
(3) Normal vector and line vector: To estimate the normal vector and the line vector, we use PCA (principal component analysis) of the points contained in the segment;
(4) Distance between scanner location and endpoints *li*: The Euclidean distance between scanner location and endpoint (first point or last point according to time attribute) of segment *i*.

**Figure 6:**
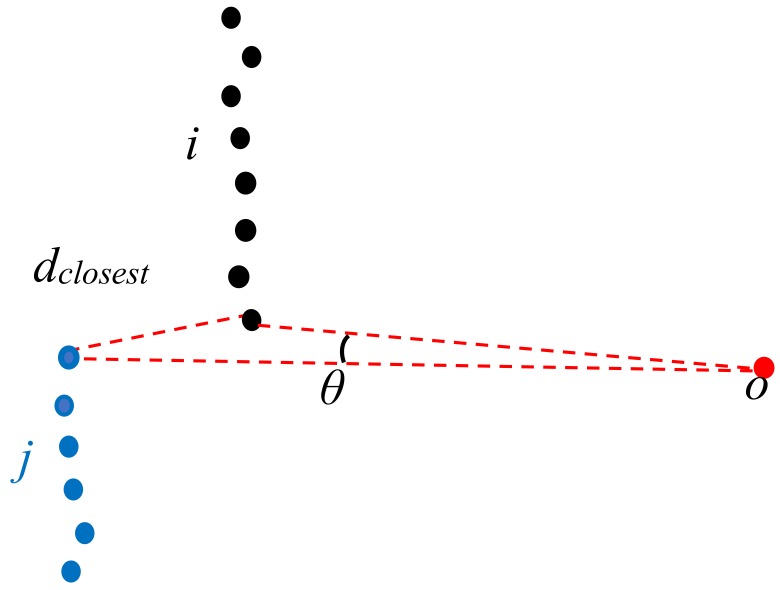
Example of a pair of segments *i* and *j* (the parameters correspond to the feature definition; point *o*: scanner location).

For a given pair of neighbour segments *i* and *j* (as shown in Figure 6), the features of this pair’s segments are shown as follows:

(1) The scanning angle between segments *θ*: The angle is intuitively illustrated in Figure 6 and defined using the scanner position as the vertex, and the nearest point of a pair of segments on the legs;
(2) Perpendicular and parallel: relations between a pair of segments can be determined based on the included angle of the normal vector and a range variable of 4° is considered in this case. Since it is rare to find perpendicular/parallel definitely.
(3) Closest distance between neighbour segments *d_closest_*: The Euclidean distance between the nearest points of segment *i* and *j*.

### 4.4. Opening Detection

Openings are essential components of indoor environments and are needed for navigation. Our opening detection approach is divided into two parts:

- Generation of opening candidates based on a single scanline (in the scanner coordinate system).
- Determination of the openings by joining multiple scanlines in the analysis (in the local coordinate system).

The openings, such as doors and windows, are usually considered holes on a plane [5]. Therefore, the basic assumption of this method is that the area of the opening will generally be between two collinear segments in a single scanline. The detection starts by finding a pair of collinear segments using a set of constraints and save the edge between the closest points of these extracted segments as an opening candidate. In order to analyse multiple extracted opening candidates, the candidates are projected onto the local coordinate system. Then, the optimal openings are determined based on geometrical relationships. This method could extract almost all windows and doors, either open or closed.

#### 4.4.1. Opening Candidate Generation

**Figure 7:**
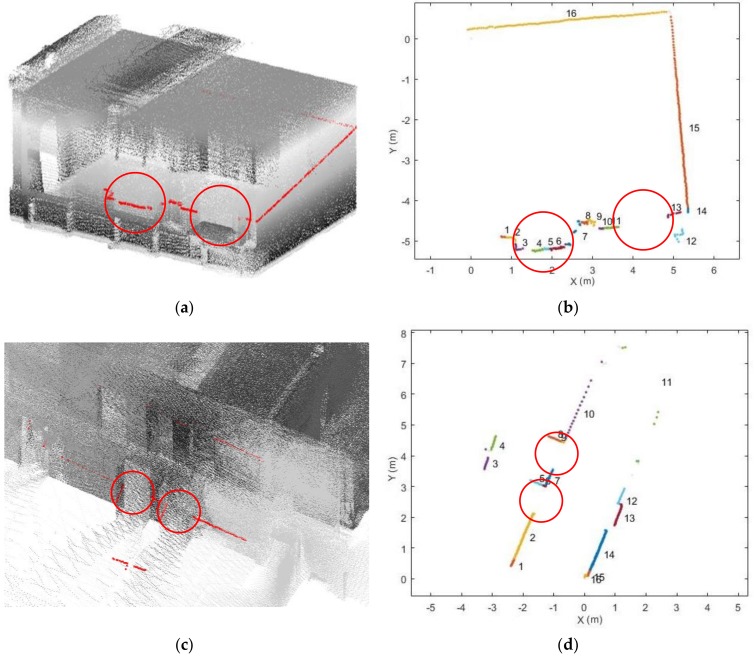

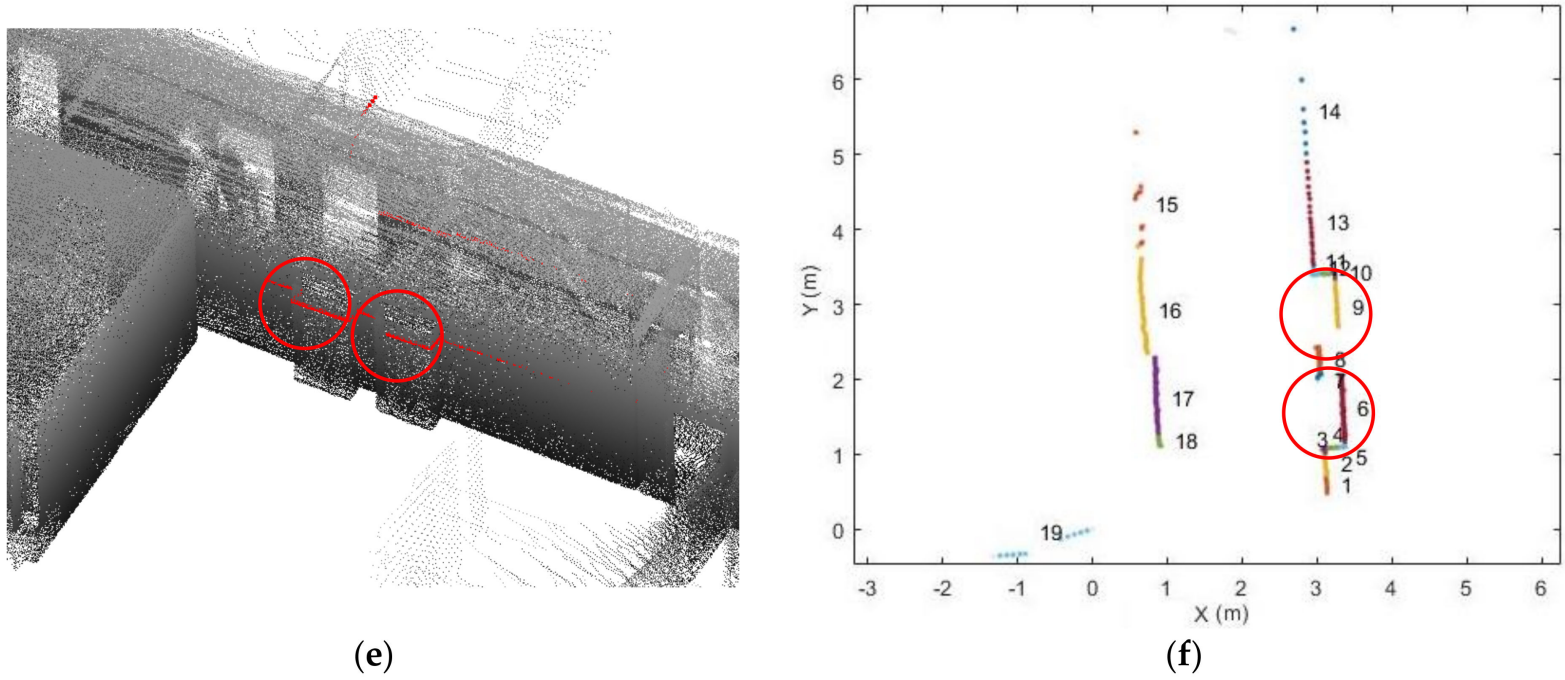
Comparison of the openings in 3D and 2D: (**a**,**b**) windows in the scanline; (**c**,**d**) open doors in the scanline; (**e**,**f**) closed doors in the scanline (red circle: opening, each segment is shown in a different colour).

As previously mentioned, openings comprise doors and windows in indoor space. Figure 7 shows some examples of how openings look like in single scanlines. The red circles in Figure 7a,b indicate two windows in the scanline. Different patterns are shown because a laser beam generally penetrates window glass, which leads to fewer points on the window [15,30]. However, in this dataset, when a laser beam penetrates a window, certain pulses will not be recorded by the scanner, whereas other beams are reflected when they bounce off the glass. Doors, either open or closed, show a more stable pattern than windows since they always contain certain segments (belonging to another space) between two colinear segments. Considering these different opening situations, a set of rules is defined based on two cases. One is there are some segments between the two collinear segments, such as the open door, the closed door and the window containing points. Another is the two collinear segments are neighbouring segments, like windows without point clouds.

Let Si and Sk be represent a pair of collinear segments. The rules for opening candidate extraction in a single scanline are defined include as follows:

- Si and Sk are neighbor segments.
  1. The scanning angle θ between these segments should be larger than $th_{angle}$.
  2. The closest distance $d_{closest}$ between Si and Sk should be larger than $th_{dynamic}$.

The angle interval *θ* generally remains stable in the same scanline. If the angular interval between neighbour segments is obviously larger than the constant value, several points may have been missed and the window area will show up as a data gap as in Figure 7b. Therefore, this first constraint is used to check whether certain points between these neighbouring segments are lost. In order to reach the object of window candidate extraction, the threshold is fixed to $th_{angle}=$ 5°. It is unnecessary to adjust this value except the dataset is acquire by another 2D scanner with different angle resolution like we used in this research.

**Figure 8:**
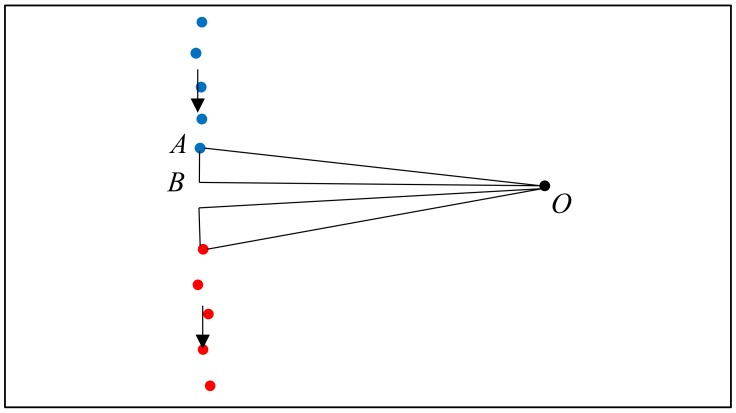
Example of a pair of segments (different colour represents different segment, black point: scanner location).

The second criterion is used to determine whether these segments are close to each other when considering that the segmentation process may generate over-segmentation result. Hence, we use the segment before to determine the distance in which would expect the next point. However, the closest distance ($d_{closest}$) between neighbor points will be affected by the distance between scanner location and the point, which explains why the dynamic threshold $th_{dynamic}$ is used in this criterion. To acquire this parameter, we construct a triangle ΔOAB in the edge points like shown in Figure 8, in which point *O* is the scanner location and point A is the edge point. The angle ∠AOB is 0.625° according to the scanning angle. The angle ∠OAB can be acquired by the line vector and the coordinates of point *O* and A in 2D space. Then, the distance between point A and B can be acquired by this triangle. Building the triangle in the two edge points and the dynamic threshold is achieved by the sum of acquired distance between point A and B.

- Si and Sk are not neighboring segments.
  1. Find the segments between Si and Sk. Let $d_{mean}$ represent the mean Euclidean distance between the centroid of these segments and the scanner position and $d_{ik}$ represent the mean distance between the closest endpoint of the two collinear segments and the scanner position. The segments between Si and Sk normally belong to another space. Therefore, $d_{mean}$ should be longer than $d_{ik}$.
  2. The closest segment should also belong to another space. Therefore, the minimum distance of the segment between the two collinear segments should be longer than $d_{ik}$.
  3. The distance of the opening segments should be longer than $d_{min}$.

If these conditions are fulfilled, we save the edge between Si and Sk as an opening candidate. Although an opening can be extracted in a single scanline, a degree of some uncertainty remains. In Figure 8, a number of wrongly extracted points are clearly present among the extracted candidates, because certain scanlines may be affected by occlusions and clutter in the indoor environment.

#### 4.4.2. Optimal Opening Determination

**Figure 9:**
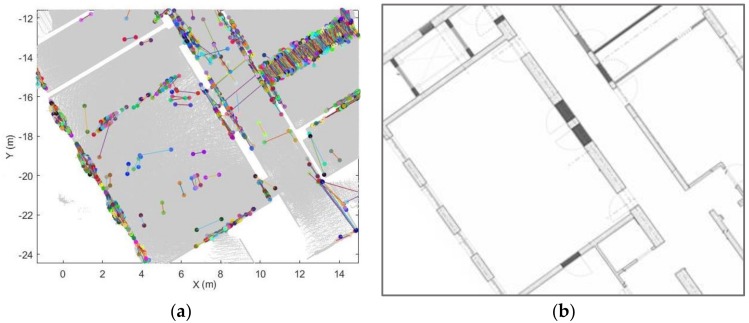
Example of extracted opening candidates in xy-plane (**a**) and it corresponding floor plan (**b**) (each opening candidate shown in a different colour).

To remove incorrect opening candidates and recover the optimal location of the opening, we analyse multiple opening candidates in this step. In the local coordinate system, the extracted opening candidates indicate the edge of the openings. The opening areas generally vertical because of the way they function, even if walls are sloped etc. Therefore, the locations of the candidates that belong to the same opening area will be close to each other in the xy-plane. As seen in Figure 9, the extracted opening candidates are concentrated in several local areas, especially areas that resemble doors and windows. Based on this assumption, we construct a circular buffer in this step. The opening $O:\left\{O_{1},\cdots ,\;O_{k}\right\}$ can be extracted if more than Nd candidates are contained within the buffer. We use the following steps to identify the optimal openings.

**Figure 10:**
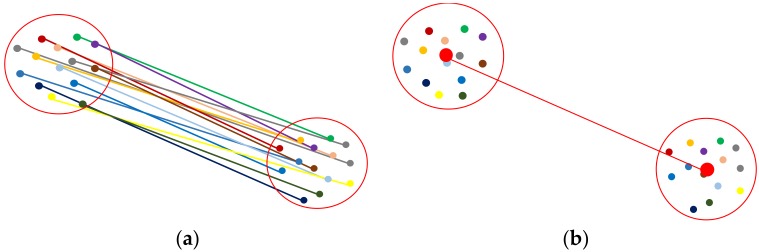
Definition of optimal opening. (**a**) opening candidates in an opening area. (**b**) extracted optimal opening position (each opening candidate shown in a different colour; red circle: buffer; red point: optimal opening position).

**Figure 11:**
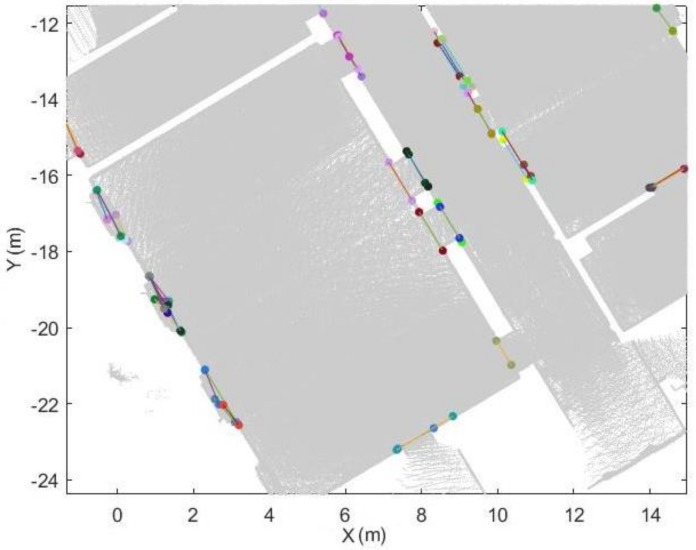
Example result of Step 2 (each opening candidate shown in a different colour).

**Figure 12:**
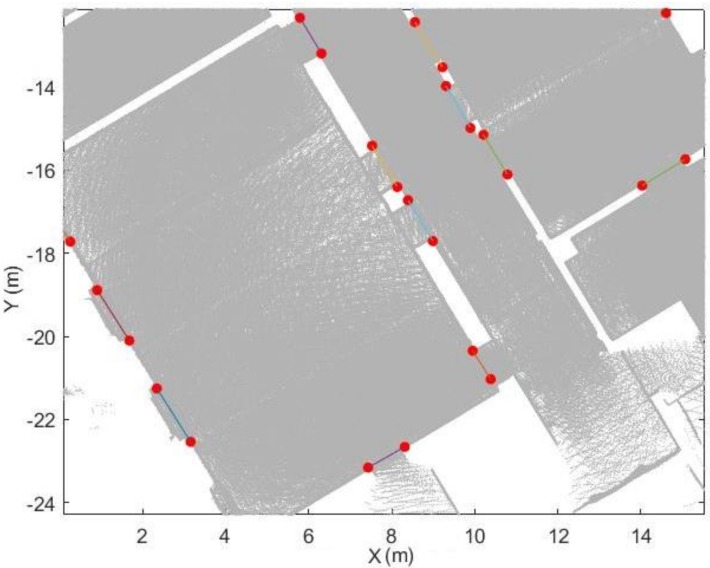
Example result of Step 3 (the final extracted openings are shown in red segments).

**Figure 13:**
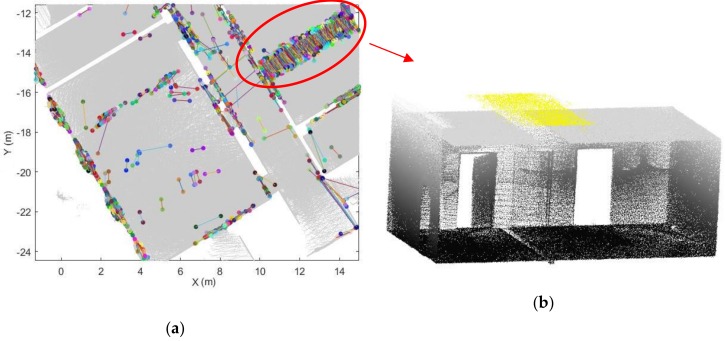
The effects of a horizontal gap **(a)** shows in xy-plane and **(b)** the corresponding 3D point clouds.

1. As mentioned above, each opening candidate contains two points. For analysis of the points on the same side, we define the point *a* as the point with the smaller x coordinate and point *b* as the point with the larger one. In order to prevent the situation where two endpoints have the same x coordinate, we set point *a* as the point with the smaller y value when the difference of the x coordinates is smaller than 0.01 m. The extracted door frame points in the xy-plane of the local coordinate system are shown in Figure 9.
2. We construct a buffer with radius r_1 for all points a and points b (red circle in Figure 10). If more than Nd points are in the same buffer for both corresponding points, we compute the location of the opening as the mean coordinates of all points in the buffers around point a and point b (red point in Figure 10b). The opening segment will be saved as these two points. This step will remove incorrect points while retaing the optimal openings. Nevertheless, the weakness of this step is that it leads to false positive results (as shown in Figure 11).
3. To refine the results of Step 2, a new buffer with radius r_2 is used to merge the extracted doorframe points. If more than one openings are contained within the same buffer, we use the mean coordinates of these openings as the location of the opening. An example result of this step shows in Figure 12.
4. The extraction method assumes that the opening plane in 3D space is oriented vertically. However, certain horizontal gaps (see yellow plane in Figure 13b) may also meet the rules of opening candidate detection. In this case, the proposed constraints will extract multiple opening candidates in this area (red circle in Figure 13a) which may lead wrong detection result. In order to solve this problem, we remove the detected openings acquired by number of point with low height variance.

### 4.5. Space Subdivision

**Figure 14:**
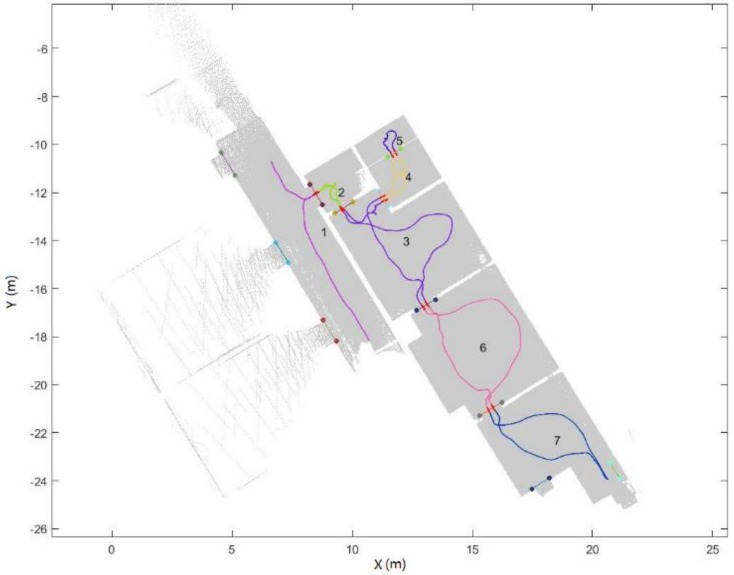
Example of trajectory subdivision result (different colours stand for different spaces).

A two-step subdivision process is defined in this section. The process starts with subdividing the trajectory into different spaces using the detected opening segments. Although this process is performed in 2D space, i.e., in the xy-plane, is can also be generalized to 3D. The first step is trajectory subdivision. If an opening intersects with the trajectory, we label this opening as a door since it is not plausible that the operator can passed through a window. Then, the defined doors are used to subdivide the trajectory. We define the trajectory points that are 0.2 m away from the door’s segment as a doorway and an example result of space subdivision is presented in Figure 14.

After the trajectory subdivision process, the point cloud can be subdivided based on the labelled trajectory.

**Figure 15:**
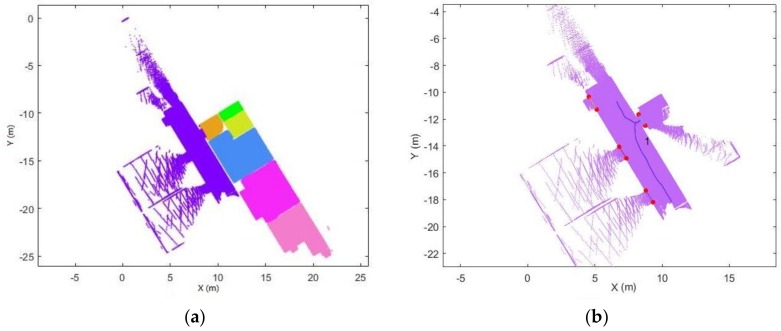
Example of the subsets of the point cloud. (**a**) all point cloud subsets. (**b**) one of the point cloud subsets (different spaces shown in different colour).

1. Each point in the trajectory corresponds to a single scanline. The point clouds can be split into several subsets based on the space label in the trajectory, as shown in Figure 15a. This process is used to accelerate the subsequent process.
2. For each point cloud subset (see Figure 15b), we construct the edge from each trajectory point to each corresponding point in the scanline. The basic assumption is that if the point belongs to the space, the edge which links the scanner position and the point will not intersect with the door segment in the xy-plane. Therefore, if the segments do not intersect with defined door segments, then the point belongs to this space.

## 5. Results and Discussion

### 5.1. Pre-Processing

**Figure 16:**
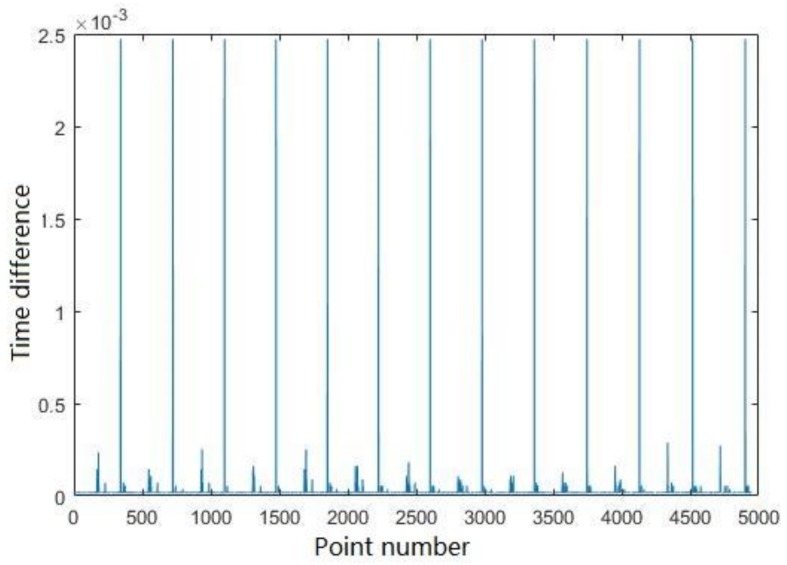
The time differences between neighbour points related to time attributes.

The pre-processing step has one parameter, $th_{time}$, which depends on the scanning system. The time difference, in general, is related to the scanning frequency. Figure 16 shows the time difference between points in the dataset with 5000 points. The vertical (*y*) axis represents the time difference and the horizontal (*x*) axis represents the sequence of input points, i.e., 1, 2 …, 5000, which supports the observation that the time difference between scanlines will be larger than the time differences between neighboring points within same scanline obviously. After analysing the plot of time differences, we set $th_{time}$ to 2.25 × 10^−3 ^s.

To improve and check the quality of the scanline, the eigenvalues (λ1, λ2, λ3) can be applied to evaluate the results of pre-processing. In point clouds, the eigenvalues represent the variance of the coordinates of all points along the eigenvector. Eigenfeatures derived from eigenvalues are commonly used to describe local geometric characteristics, and they can present, check and prove whether the local geometry is planar or spherical [31]. The basic assumption of the scanline is as follows: if all points belongs to the same scanline, then they should lie on the same plane. In order to describe the geometric characteristics and indicate whether the geometry of an extracted single scanline is planar, eigenvalues are applied to evaluate the results of pre-processing.

Table 3 shows the mean and variance of the eigenvalues. As the table illustrates, the variance along the plane normal is estimated by the smallest eigenvalue. The mean and variance of λ1 is almost equal to zero, which means that almost all extracted scanlines lie on the same plane.

### 5.2. Opening Detection

**Figure 17:**
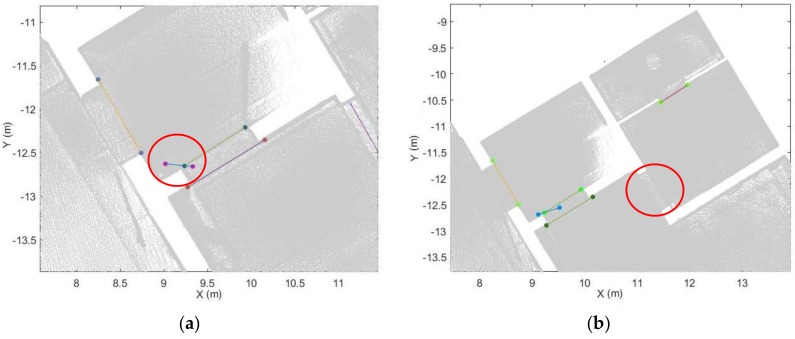
Influence of the parameters r_1 and Nd. (**a**) $\;r_{1}=0.5\;\left(m\right)$ and Nd=6; and (**b**) $r_{1}=0.3\left(m\right)$ and Nd=8.

**Figure 18:**
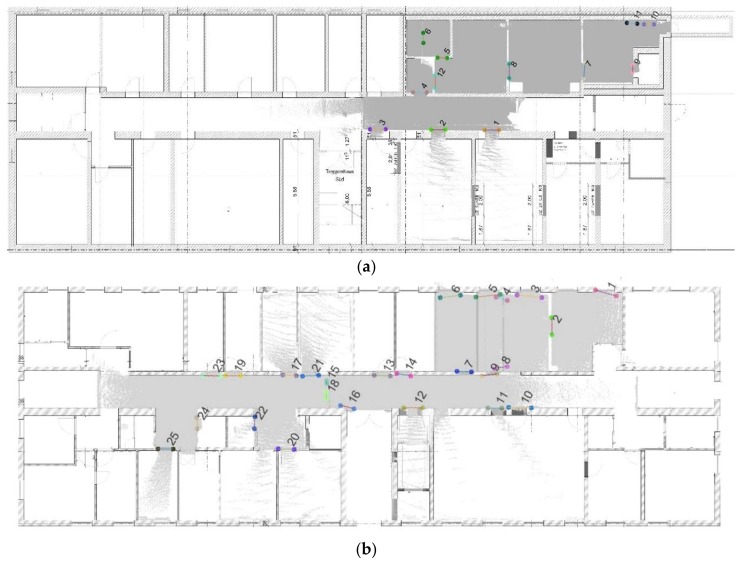
Visual comparison of the opening extraction results and ground plan (**a**) extracted openings in dataset 1; and (**b**) extracted doors in dataset 2 (opening segments are shown in different colours).

Four parameters are included in the opening detection step: $\;d_{min}$, r_1, r_2 and Nd. The minimum distance of the opening segments ($d_{min}$) is used to remove short opening segments. This parameter is fixed to $d_{min}=0.7\;m$ based on experimental results. This parameter is unnecessary to adjust since the minimum width of an opening will not change based on the scanner or dataset. The radius (r_1) of the buffer and the minimum number (Nd) of the segments have the greatest impact on the quality of opening detection. Large values of r_1 will lead to a greater number of candidates under consideration, which means that they are much more easily affected by the wrong opening candidates (an example is shown by the red circle in Figure 17a). As for the minimum point number Nd, a small value may remove correct openings (an example is shown by the red circle in Figure 17b). Therefore, these two parameters were fixed to $r_{1}=0.3\;m$ and Nd=6 based on the experiment. The circle buffer of radius r_2 used to remove over-detected opening segments and merge closely located was set to 0.5 m in this research. Then, the opening detection results shows in Figure 18.

**Figure 19:**
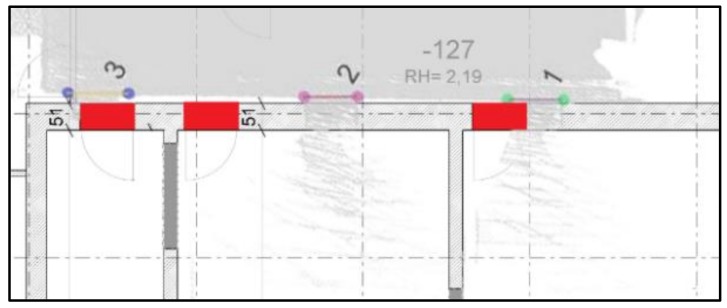
Comparison of the results obtained using opening detection and the ground plan (detail in Figure 17a, red: opening location in the ground plan).

**Figure 20:**
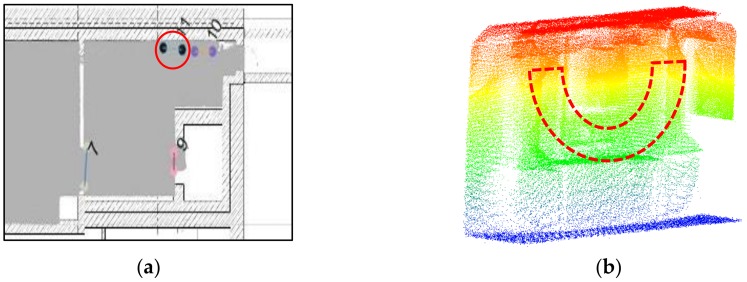
Comparison of the detection results (**a**) with point clouds (**b**).

Using the manually registered ground plan as a reference, a visual analysis is conducted to determine whether the openings are correctly and completely extracted. As shown in Figure 18a, we detect 11 openings in the dataset, and openings 10 and 11 are misdetected, although others match the corresponding doors in the ground plan. This method can detect closed doors (such as doors 3 and 9 in Figure 18a and doors 13, 14, 19 and 23 in Figure 18b), but it is noticed the detection of closed doors is based on the door frame’s geometry so it will not detect closed doors that are co-planar to the wall. Moreover, doors 1 and 2 may not well match the location (as shown in Figure 19), which can be explained as follows: the SLAM results include errors or the ground plan is incorrect. Besides, the two mis-detected doors (doors 10 and 11 in Figure 18a) are also shown in Figure 19a, and certain openings are detected that resemble windows in a basement. The corresponding point cloud show that certain parts of this wall (red part in Figure 20b) are concave. This error demonstrates that the door detection method may be affected by indoor objects that present similar geometric structures as the openings.

**Figure 21:**
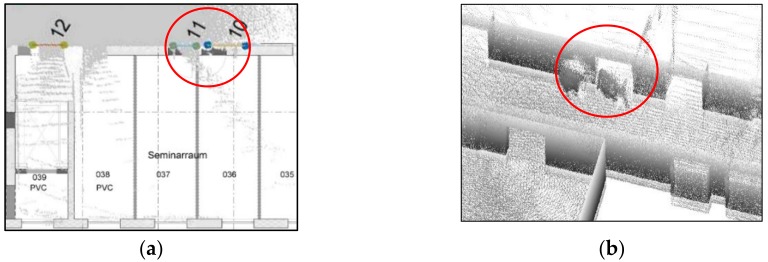
Mis-detected door in the ground plane (**a**) xy-plane (**b**) and 3D point clouds (detail in Figure 17b).

**Figure 22:**
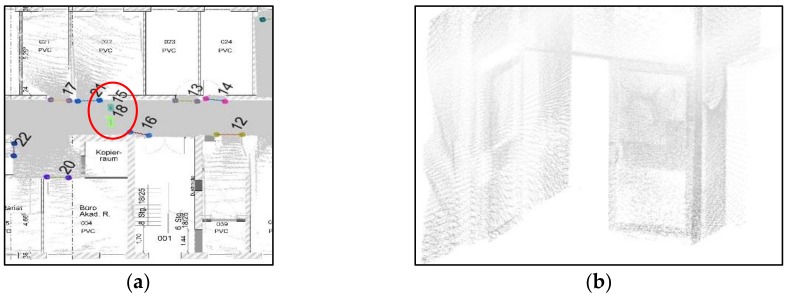
Point cloud of the double-door in the corridor area.

As shown in Figure 18b we detected 25 openings in this dataset, while some openings present in the ground plan were not detected, which is partly explained by occlusion. For example, the door between door 11 and 12 in Figure 21a marked by the red circle is not detected because this door is occluded by another object. Therefore, the expected pattern in a single scanline is not evident (see Figure 21b). Another reason for the lack of detection is the low number of opening candidates. If the number of candidates within the same buffer does not meet the defined criterion, then these candidates will be ignored. Moreover, doors 8 and 9 in Figure 18b do not fit well with the wall, with multiple possible reasons. First, a poor segmentation result may occur. For example, if the points in a corner are discarded in the segmentation process, then accurately-acquired points will not be used in the opening detection step. Second, points may be sparse within the scanline. Interestingly, the double door in the corridor area is extracted as doors 15 and 18 in Figure 22a because the double door is depicted as one open door and one closed door (as shown in Figure 22b) during data acquisition. Moreover, sufficient opening candidates in the closed glass door are extracted, and they could be used for opening detection. In short, the openings in both datasets can correctly and completely detect almost all the openings, even for certain doors (such as doors 19 and 23 in Figure 18b) that are close to each other.

### 5.3. Trajectory Subdivision

**Figure 23:**
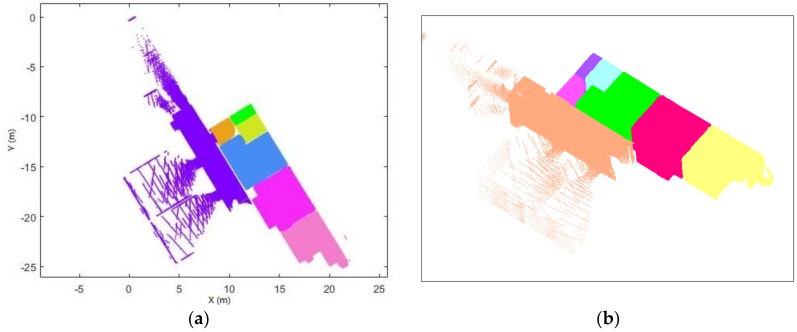
Space subdivision result in 2D (**a**) and 3D (**b**) view (different space shown in different colour).

Figure 14 provides an overview and the subdivision results of the trajectory. The result of the space subdivision is shown in Figure 23. Environment are correctly subdivided into different space. However, it cannot subdivide the space that operator did not entered.

## 6. Conclusions and Future Work

In this study, a novel method was designed to detect openings as well as subdivide indoor spaces based on scanline analysis. The proposed method uses a set of constraints to analyse the geometric information of the scanlines in the local area. For the opening detection, we detect openings in indoor environments and analyse the results of the proposed method. The main limitation of this method is that it only related to the geometric characteristics of a single scanline. Hence, it depends on the environment and the quality of the acquired point cloud. Additionally, several errors might be observed because glass doors and unconventional indoor structures were not considered in this method. The space subdivision results show that most of the space was correctly subdivided. Nonetheless, if the operator enters a room through a door and leaves through another door, excess subdivisions may be observed. Moreover, certain spaces that the operator did not enter could not be subdivided in the point clouds. These limitations are directly linked to the basic assumptions that subdivision based on the defined doors. Because the doors were defined by the intersection between opening segments and trajectory, and the space subdivision is based on the defined doors.

The proposed method analyses the coordinates of candidates in the xy-plane so far instead of their 3D distribution. Future work will focus on separating doors and windows based on opening detection, which has the potential to directly detect these features and improve the quality of space subdivision.

## Figures and Tables

**Table 1:** Datasets used in this research.

Dataset	Points	Scan Lines	Duration(s)
Dataset 1, Point Cloud	477,5931	15,036	147.167
Dataset 1, Trajectory	14,717	/	147.158
Dataset 2, Point Cloud	2,999,507	9309	91.719
Dataset 2, Trajectory	9172	/	91.709

**Table 2:** Technical characteristics of the ZEB-REVO according to the manufacturer datasheet.

Point Per Scan Line	Field of View	Scan Rate	Angle Resolution
432(0.625° interval)	270° × 360°	100 lines/s 43,200 points/s	0.25°

**Table 3:** Mean and variance of all the eigenvalues of the scanlines.

	Dataset 1			Dataset 2		
	λ1	λ2	λ3	λ1	λ2	λ3
Mean	1.31 × 10^−5^	0.78	2.02	3.05 × 10^−5^	2.17	4.92
Variance	9.73 × 10^−10^	3.29	3.29	2.56 × 10^−9^	1.75	10.32
